# Dihydrotestosterone Regulates Hair Growth Through the Wnt/β-Catenin Pathway in C57BL/6 Mice and *In* *Vitro* Organ Culture

**DOI:** 10.3389/fphar.2019.01528

**Published:** 2020-01-23

**Authors:** Xianyan Chen, Ben Liu, Ying Li, Le Han, Xin Tang, Wenjia Deng, Wei Lai, Miaojian Wan

**Affiliations:** ^1^Department of Dermatology, The Third Affiliated Hospital of Sun Yat-sen University, Guangzhou, China; ^2^Department of Dermatology, Hexian Medical Affiliated Hospital of Southern Medical University, Guangzhou, China; ^3^Department of Dermatology, Guangzhou Eight People’s Hospital, Guangzhou, China

**Keywords:** hair follicle, dihydrotestosterone, hair growth, Wnt/β-catenin pathway, β-catenin activator, androgenetic alopecia

## Abstract

Dihydrotestosterone (DHT) is the most potent androgen that regulates hair cycling. Hair cycling involves cross-talk between the androgen and Wnt/β-catenin pathways. However, how DHT regulates hair follicle (HF) growth through the Wnt/β-catenin pathway has not been well investigated. This study aimed to investigate the roles of DHT in hair growth *in vivo* and *in vitro*. Human scalp HFs were treated with different concentrations of DHT (10^-5^, 10^-6^, 10^-7^, 10^-8^, and 10^-9^ mol/L) for 10 days. The effects of DHT on hair shaft elongation, the proliferation of hair matrix cells, and the levels of β-catenin, GSK-3β, and phosphorylated GSK-3β (ser9) were evaluated in the cultured HFs. The effects of DHT were further investigated in C57BL/6 mice. Moreover, the growth of cultured human HFs was observed after interfering with the β-catenin pathway through inhibitors or activators in the presence or absence of DHT. We found that different concentrations of DHT had different effects on human HFs *in vitro* and C57BL/6 mice. At 10^-6^ mol/L, DHT inhibited HF growth and β-catenin/p-GSK-3β expression, whereas 10^-7^ mol/L DHT induced HF growth and β-catenin/p-GSK-3β expression. In addition, a β-catenin inhibitor (21H7) inhibited HF growth *in vitro*, while a β-catenin activator (IM12) promoted HF growth *in vitro* and antagonized the inhibition of HFs by high levels of DHT. These results suggest that DHT plays a pivotal role in region-specific hair growth, which may be related to the Wnt/β-catenin pathway.

## Introduction

Many hormones participate in the regulation of hair follicle (HF) growth and cycling, of which androgens are the most representative(Al-Nuaimi et al., 2010; Inui and Itami, 2013). Androgens have a profound effect on the growth of human scalp and body hair, such as promoting beard growth but leading to hair loss in androgenetic alopecia (AGA) in males (Randall et al., 2000; Randall, 2007). AGA results from an abnormal sensitivity of balding scalp HFs to circulating testosterone (T). In 1996, Itami showed that a higher level of 5a-reductase type 2 (5aR2) was found in balding scalp HFs than in occipital scalp HFs, which can convert T to the more potent DHT (Itami et al., 1996). This finding indicates that balding scalp HFs have a higher level DHT than nonbalding scalp HFs, which is consistent with the results of a double-blind study showing that DHT levels were significantly higher in a bald scalp than in a hair-containing scalp (Dallob et al., 1994). Once 5aR2 is inhibited by finasteride, an oral drug that can decrease both serum and scalp skin DHT levels, AGA progression is delayed (Drake et al., 1999; Price, 1999). These findings suggest that androgens are required to maintain AGA balding.

As mentioned before, androgens stimulate hair growth in many areas and have paradoxical effects on human HFs. They cause males to have more hair on the face and promote pubic and axillary hair development in both sexes, while often causing balding in the same individual (Hamilton, 2010; Ceruti et al., 2017; Miranda et al., 2018). However, androgens are essential for hair growth. Male pseudohermaphroditism patients show nearly no beard growth or AGA hair loss because of a lack of 5aR2, which suggests that DHT is necessary for beard growth (Andersson et al., 1991; Adachi et al., 2000; Jakubiczka, 2015). Moreover, androgens regulate hair growth in both males and females. There are a number of female subjects with abnormal hair growth, such as hirsutism in polycystic ovary syndrome (PCOS) patients, hair loss in females affected by AGA and female pattern hair loss, and these conditions are closely related to androgens (Blumepeytavi and Hahn, 2008; Lizneva et al., 2016). However, the mechanisms by which androgens have simultaneous but different effects on one organ, the HF, in different areas of the body in the same individual have not been well studied.

The Wnt/β-catenin pathway is known to positively affect mammalian HF growth and cycling (Andl et al., 2002; Ito et al., 2007). After activation, β-catenin accumulates in the cytoplasm and then translocates to the nucleus, where it interacts with Lef/Tcf transcription factors to regulate the expression of genes responsible for HF growth. The activity of β-catenin, the key molecule in the Wnt/β-catenin pathway, can be suppressed by glycogen synthase kinase-3β (GSK-3β), which is inhibited by phosphorylation. DHT abrogates the ability of dermal papilla cells (DPCs) from patients with AGA to induce HF stem cell differentiation *via* inhibition of the Wnt/β-catenin pathway in DPCs, which involves inhibiting GSK-3β activity (Mulholland et al., 2005). Therefore, we hypothesized that the Wnt/β-catenin pathway is essential to HF growth regulation by DHT.

The present study shows that the impact of DHT on HF growth and cycling varies at different concentrations by interacting with the Wnt/β-catenin signaling pathway. We provide evidence that activation of the Wnt/β-catenin pathway can weaken the negative influence of high-dose DHT on HFs.

## Materials and Methods

### HFs Culture

Occipital nonbald human scalp skin was donated from males and females undergoing hair transplant surgery. HF donors were selected randomly from among patients who did not take any antiandrogens in the past 3 months and who did not have inflamed scalp skin. All 12 volunteers (men and women, ages 20–45 years) signed the informed consent form for participation in this study.

Anagen VI HFs (Leirós et al., 2012) were isolated by microdissection with ophthalmic forceps and a scalpel blade, and the follicles were separated into a single follicle under a dissecting microscope (Nikon, Japan). Fat was removed carefully, and HFs were cut at the dermo-subcutaneous fat interface. Isolated HFs were maintained individually in 24-well plates with 500 μl serum-free Williams’ E medium (Gibco, USA), supplemented with 2 mM L-glutamine (Gibco, USA), 2 mM HEPES (Gibco, USA), 10 mg/L transferrin (MP Biomedicals, USA), 10 μg/L sodium selenite (MP Biomedicals, USA), 10 μg/L hydrocortisone (MP Biomedicals, USA), 10 mg/L insulin (Sigma-Aldrich, USA), and 1% antibiotics (Gibco, USA). HFs were maintained at 37°C in an atmosphere of 5% CO_2_/95% air.

### DHT Treatment

After 24 h, the live HFs that grew 0.3–0.5 mm were cultured with DHT (10^-5^~10^-9^ mol/L, MP Biomedicals, 521-18-6, purity≥98% (HPLC), USA) for 10 days. The hair shaft length was measured every 2 days, and the mean growth rate of the hair shaft was calculated. At 10 days, partially isolated HFs cocultured in 10^-6^ mol/L or 10^-7^ mol/L DHT were analyzed for the proliferation of hair matrix cells by Ki-67 staining. Ethanol (0.1%) dissolved in serum-free Williams’ E medium served as the vehicle in the negative control group.

### Hair Growth *In Vivo*

Male 7-week-old C57BL/6 mice (n = 40) purchased from Nanjing University Model Animal Research Institute (Nanjing, China) were housed in plastic cages in a room with controlled temperature (22°C ± 1°C) and humidity (55 ± 15%). Mice were maintained on a regular pellet diet with access to fresh water. After acclimatization for 7 days, all mice were depilated with 6% sodium sulfide solution in the dorsal area (approximately 2 cm in width and 4 cm in length) before being anesthetized. Mice were randomly divided into 4 groups administered different treatments: negative control group, 40% DMSO dissolved in saline solution; experimental groups, DHT (10^-6^, 10^-7^, and 10^-8^ mol/L). All mice were administered 200 μl solution to the test area every day for 17 days, and the back skin of these mice was observed and photographed. After depilation at day 14, the mouse back skin was isolated to examine histological features by hematoxylin and eosin staining and related-proteins expression by Western blot. The individual mouse skin samples were fixed in 4% paraformaldehyde (Servicebio, China) for 24 h, cut into 3-μm sections with a pathology microtome (Leica, German), and stained with hematoxylin and eosin; the histological morphology of the skin was examined by light microscopy (NIKON, Japan).

### Western Blot

Here, Nuclear and Cytoplasmic Protein Extraction Kit (Beyotime, #P0028, Shanghai, China) was employed to isolate the nucleoproteins of the back skin or cell lysis buffer to lyse the total proteins; after that, the concentrations of these nucleoproteins and total proteins were measured by a micro BCA protein assay kit (Pierce, Irvine, CA, USA). Afterwards, a total of 50 μg per lane of nucleoproteins or total proteins was added into the well of sodium dodecyl sulfate-polyacrylamide gel electrophoresis gels (Invitrogen), and immunoblotting was performed using an anti-β-catenin (1:5000, Abcam, ab32572, USA), anti-GSK3β (1:5000, Abcam, ab32391, USA), and anti-p-GSK3β (1:500, Abcam, ab131097, USA) antibodies. Lamin B and GAPDH levels were used for the normalization of nucleoproteins or total proteins, respectively. Finally, scanned and quantified the protein bands using a ChemiDoc image analysis system (Bio-Rad Laboratories). The relative protein levels were determined by calculating the ratio of the value of the protein band of interest to that of the corresponding GAPDH or Lamin B band.

### Immunofluorescence

The cultured HFs were fixed in 4% paraformaldehyde (China) for 24 h and then cut into 3-μm sections. After dewaxing, the sections were soaked with 3% H_2_O_2_ for 10 min and preincubated with normal goat serum (DAKO, Denmark) for 30 min. Then, the paraffin sections were incubated with primary antibodies against Ki67 (1:200, Abcam, ab8191, USA), β-catenin (1:200, Abcam, ab32572, USA), GSK3β (1:200, Abcam, ab32391, USA), p-GSK3β (1:100, Abcam, ab75814, USA), or androgen receptor (AR, 1:600, CST, #5153, USA) at 4°C overnight. Afterwards, the sections were incubated with secondary FITC- or CY3-conjugated goat anti-mouse/rabbit antibody for 50 min. Next, the sections were stained with 10 μg/ml DAPI (Abcam, USA). These sections were observed under an inverted fluorescence microscope (Nikon, Japan), and images were collected (FITC green excitation wavelength 465–495 nm, emission wavelength 515–555 nm; CY3 red light excitation wavelength 510–560, emission wavelength 590 nm). The relative expression levels of Ki67, β-catenin, GSK3β, and p-GSK3β were analyzed by the Image pro-plus 6.0 software: first of all, the immunofluorescence image is converted into a black and white image; then the integrated optical density (IOD) of the positive region is analyzed; afterwards, calculate the area of positive region (AREA); finally, mean density is obtained as follows: Mean density = IOD/AREA.

### 21H7 and IM12 Treatment

21H7 is a selective inhibitor of the Wnt/β-catenin pathway that destabilizes β-catenin, while IM12 is an activator of the Wnt/β-catenin pathway that significantly increases β-catenin levels (Xu et al., 2015). After 24 h, the live HFs that grew 0.3–0.5 mm were cultured with IM12 (50, 100, or 500 nM; Sigma-Aldrich, #SML0084, purity ≥ 98% (HPLC), USA) or 21H7 (1, 2, or 4 μM; Sigma-Aldrich, #SML0570, purity ≥ 98% (HPLC), USA). Hair shaft length was measured every 2 days, and the mean growth rate of the hair shaft was calculated. Partially isolated HFs were cultured in DHT (10^-6^ mol/L), IM12 (500 nM) or 10^-6^ mol/L DHT and 500 nM IM12. After 10 days of culture, the HFs were collected to analyze β-catenin expression using an immunofluorescence assay.

### Statistical Analysis

Data were statistically analyzed and graphed using GraphPad Prism 5 (GraphPad Software, USA). All results are presented as the mean ± standard deviation. Statistically significant differences between groups were determined by Student’s *t*-test. Multiple comparisons among ≥3 groups were performed using one-way ANOVA followed by the Bonferroni *post hoc* test. The nonparametric Mann-Whitney U test was used if data were not normally distributed. *P* < 0.05 was considered statistically significant.

## Results

### Effects of DHT on Human HF Growth *In Vitro*

**Figure 1:**
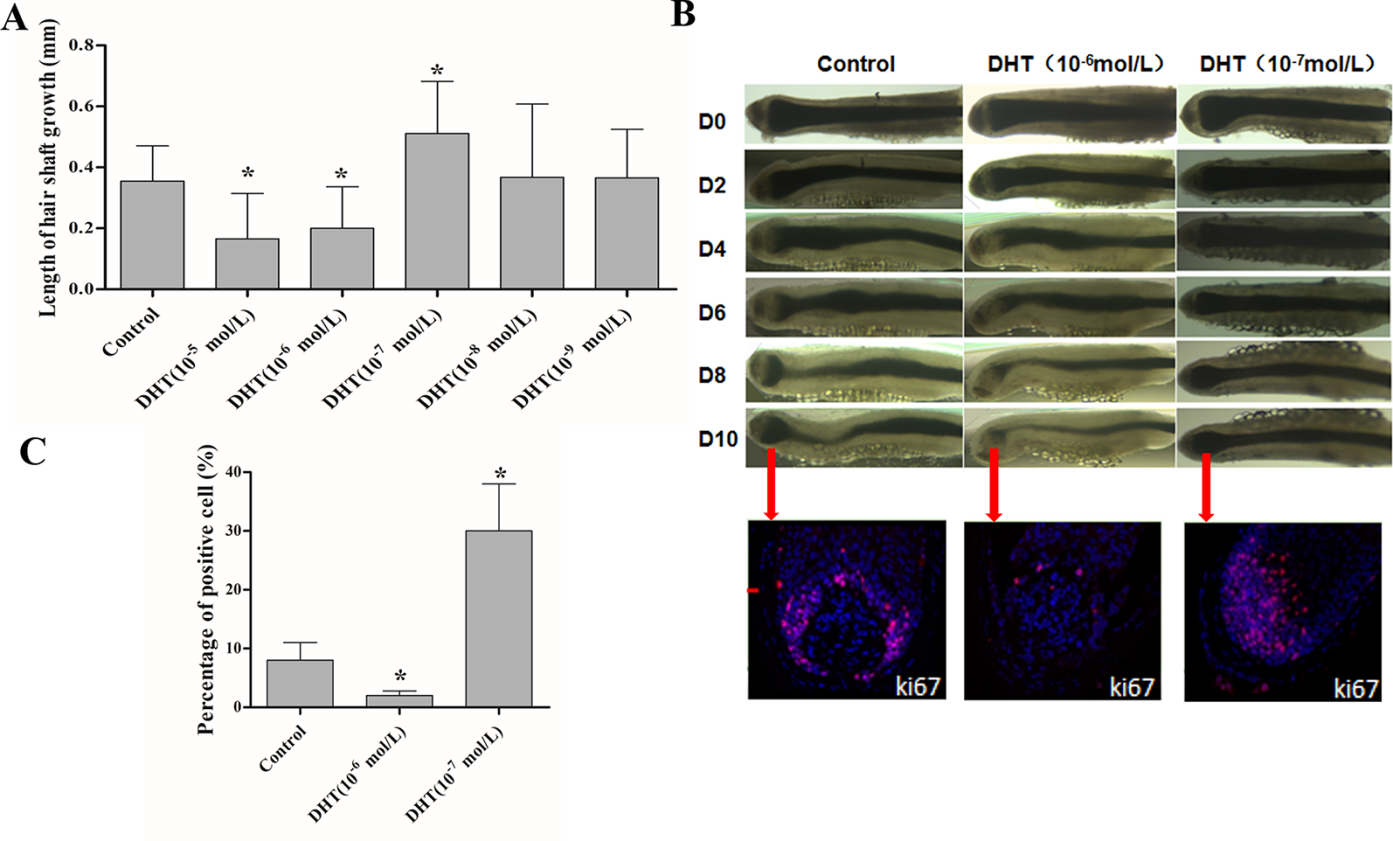
Effects of different concentrations of dihydrotestosterone (DHT) on hair shaft growth of human HFs *in vitro*. Isolated anagen human HFs were cultured with DHT at various concentrations or vehicle for 10 days. **(A)** Mean growth rates of hair shafts. Hair shaft length was measured every 2 days (n = 18). **(B)** Changes in hair bulb morphology during the 10-day experimental period and immunofluorescence staining of Ki67 in HFs cultured with various concentrations of DHT (0, 10^-6^ and 10^-7^ mol/L) at day 10. **(C)** Percentages of Ki-67-positive cells in the matrix area (n = 6). The data are presented as the mean ± SD. Each bar represents the mean of three independent experiments performed in triplicate. Compared with the vehicle-treated control group, ^*^P < 0.05.

First, we evaluated the effects of DHT on human HF growth based on growth rate, morphology of the hair matrix (HM) and the dermal papilla (DP), and HF proliferative activity. As shown in **Figure 1A**, 10^-5^ and 10^-6^ mol/L DHT inhibited HF growth, while 10^-7^ mol/L DHT induced HF growth. Additionally, compared with control-treated HFs, HFs had a considerably thinner HM, a more onion-shaped DP, less melanin content and a lower percentage of Ki-67-positive cells by exposing to 10^-6^ mol/L DHT. While HFs which was treated with 10^-7^ mol/L DHT showed a HM with a larger DP volume, maximal melanin content and a significantly higher number of Ki-67-positive cells. (**Figures 1B, C**). Furthermore, the HFs after treated with 10^-6^ mol/L DHT entered the catagen phase much faster than the control-treated HFs, and the HFs treated with 10^-7^ mol/L DHT opposite. Therefore, in the following study, we chose 10^-6^ mol/L as the optimal concentration of DHT that inhibits human HF growth, which is close to the circulating concentration of human DHT.

### Effects of DHT on Hair Regeneration in C57BL/6 Mice

**Figure 2:**
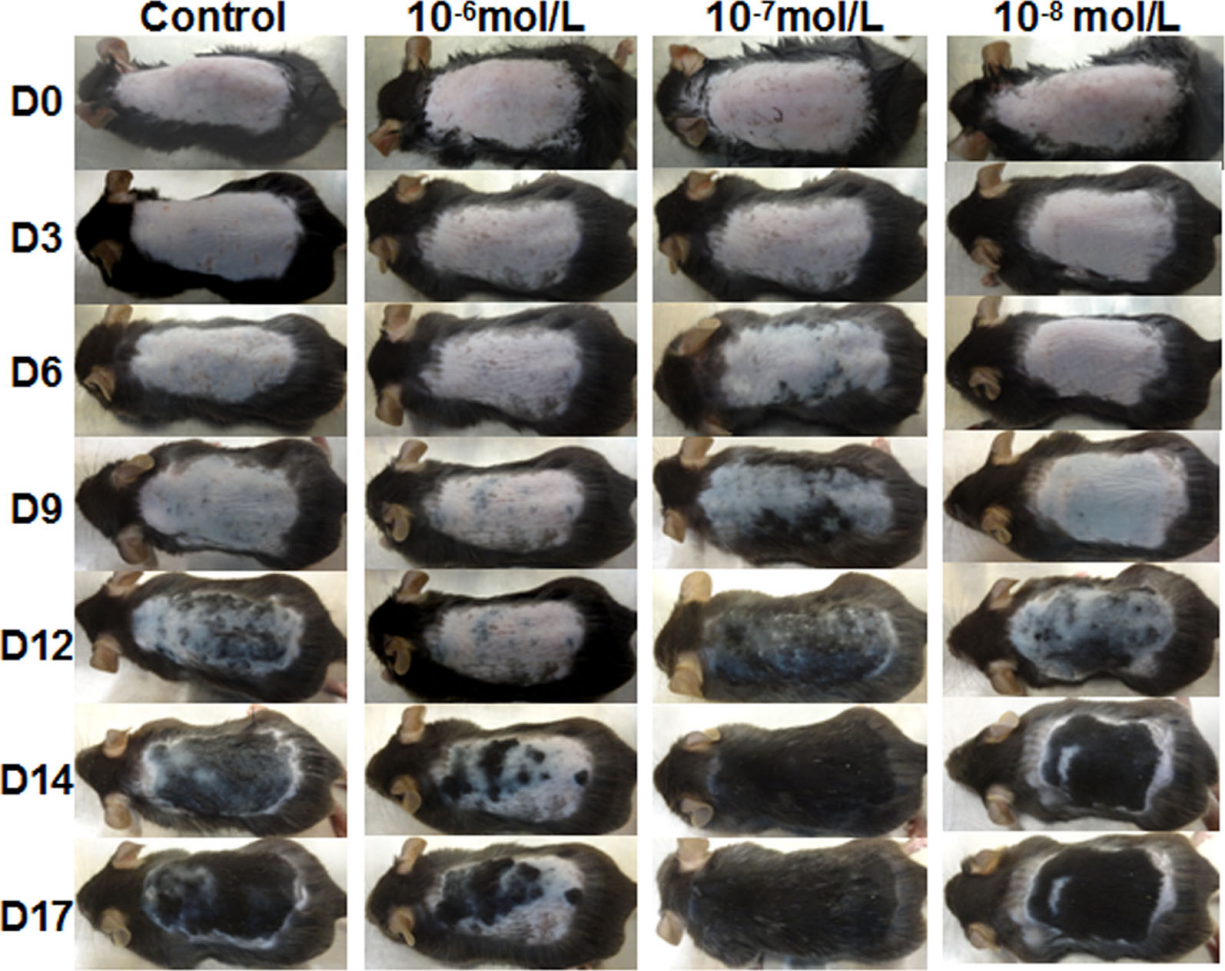
Effects of topical dihydrotestosterone (DHT) on hair regeneration in C57BL/6 mice. The back skin was treated with DHT (10^-6^, 10^-7^, or 10^-8^ mol/L) every day for 18 days in the DHT groups, while the vehicle-treated control group was treated with 40% DMSO. The back skin was photographed at days 0, 3, 6, 9, 12, 14, and 17 after treatment initiation.

**Figure 3:**
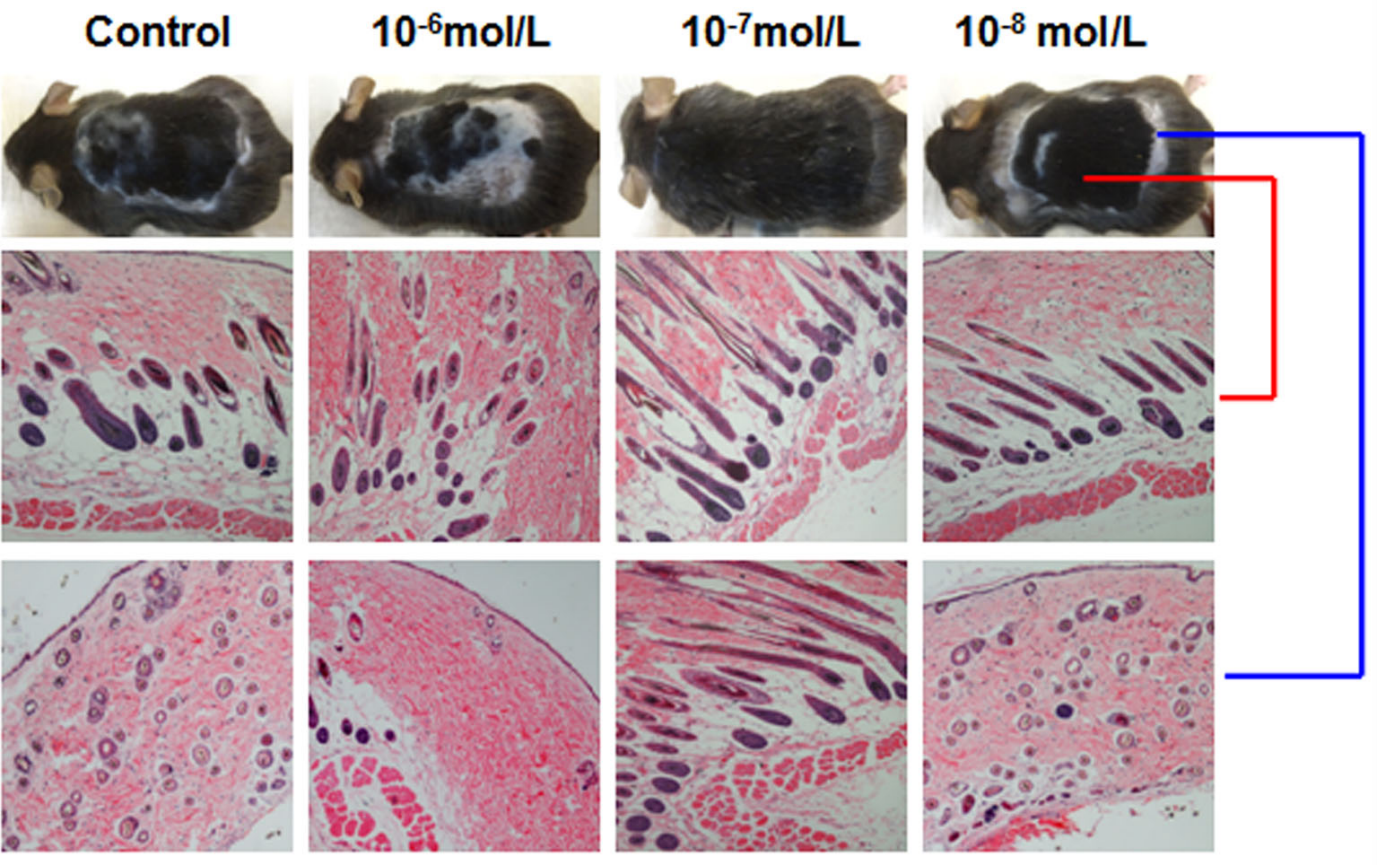
Effects of different concentrations of dihydrotestosterone (DHT) on hair follicle morphology in C57BL/6 mice. The back skin of C57BL/6 mice at 18 days after treatment was shaved and then stained with hematoxylin and eosin. Representative HE staining images from mice treated with different concentrations of DHT are shown. The red line refers to back skin, while the blue line represents gray skin.

Then, we investigated the effects of DHT on HF cycling *in vivo*. Depilation induces HFs to enter the next growth cycle synchronously in C57BL/6 mice. Depilated dorsal mouse skin is pink in the telogen phase, is gray when anagen is initiated, and then gradually darkens. As shown in **Figure 2**, the control group and the 10^-8^ mol/L DHT group exhibited light gray skin at day 9 after depilation, the low-dose DHT (10^-7^ mol/L) group had darker skin than the other groups, and the 10^-6^ mol/L DHT group still exhibited pink skin at day 9. At day 12, hair regeneration was obvious in the low-dose DHT (10^-7^ mol/L) group compared with the control group, while the skin of mice in the high-dose DHT (10^-6^ mol/L) group remained pink at this time point. HE staining (**Figure 3**) indicated that more hair shafts were observed in the low-dose DHT (10^-7^ mol/L) group than in the other groups. These results suggest that the effects of DHT on HF growth depend on its concentration: the entry of HFs into the anagen phase in C57BL/6 mice is induced by 10^-7^ mol/L DHT but inhibited by 10^-6^ mol/L DHT.

### DHT Regulates HF Growth Through the Wnt/β-Catenin Pathway

**Figure 4:**
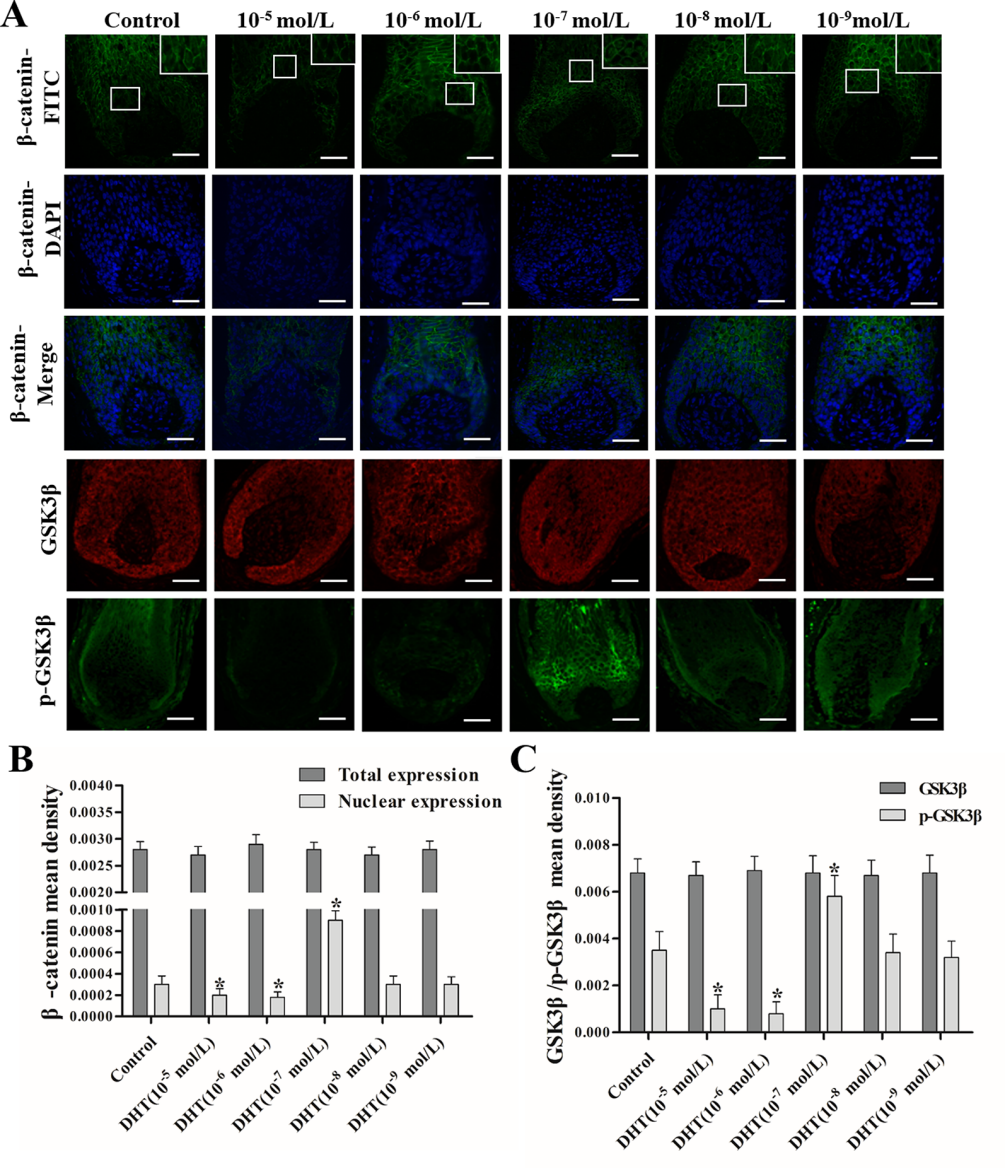
Effects of different concentrations of dihydrotestosterone (DHT) on the expression of β-catenin, GSK3β, and p-GSK3β (ser9) in human HFs. The hair follicles (HFs) were fixed and labeled with anti-β-catenin, anti-GSK3β, and anti-p-GSK3β (ser9) primary antibodies, followed by incubation with fluorescent secondary antibodies. **(A)** The HFs were visualized: green, β-catenin and p-GSK3β (ser9); red, GSK3β (magnification: 200× and 400×). **(B**, **C)** The mean fluorescence intensity of β-catenin, GSK3β, and p-GSK3β (ser9) in the matrix area. The data are presented as the mean ± SD (n = 6). Each bar represents the mean of three independent experiments performed in triplicate. Compared with the control group, ^*^P < 0.05. Scale bar 50 μm (200×).

**Figure 5:**
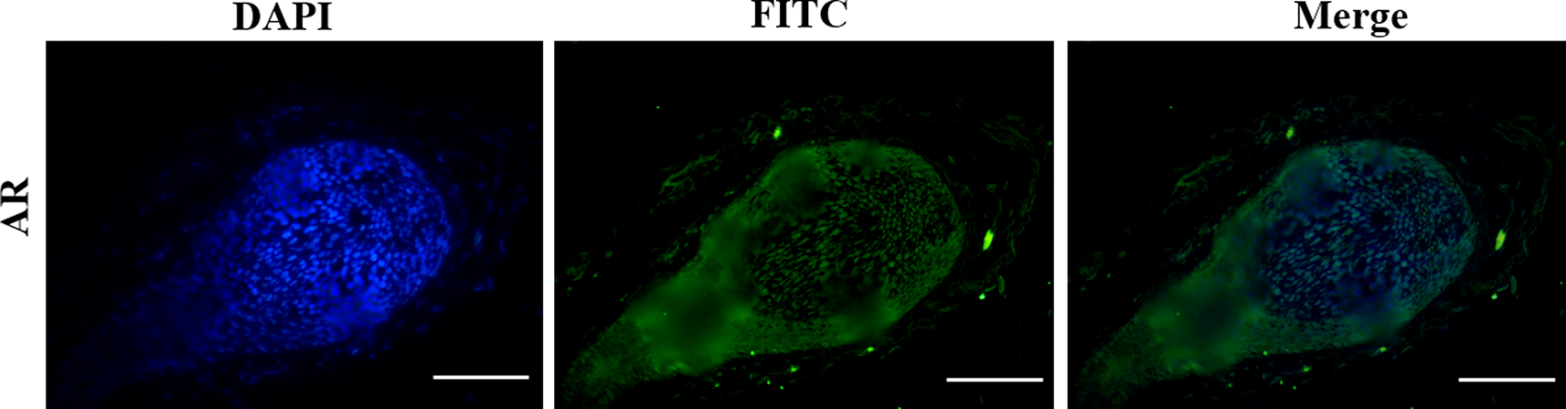
The expression pattern of AR in the normal human hair follicles (HFs). AR is mainly expressed in dermal papilla cells and hair matrix cells. Scale bar 50 μm (200×).

**Figure 6:**
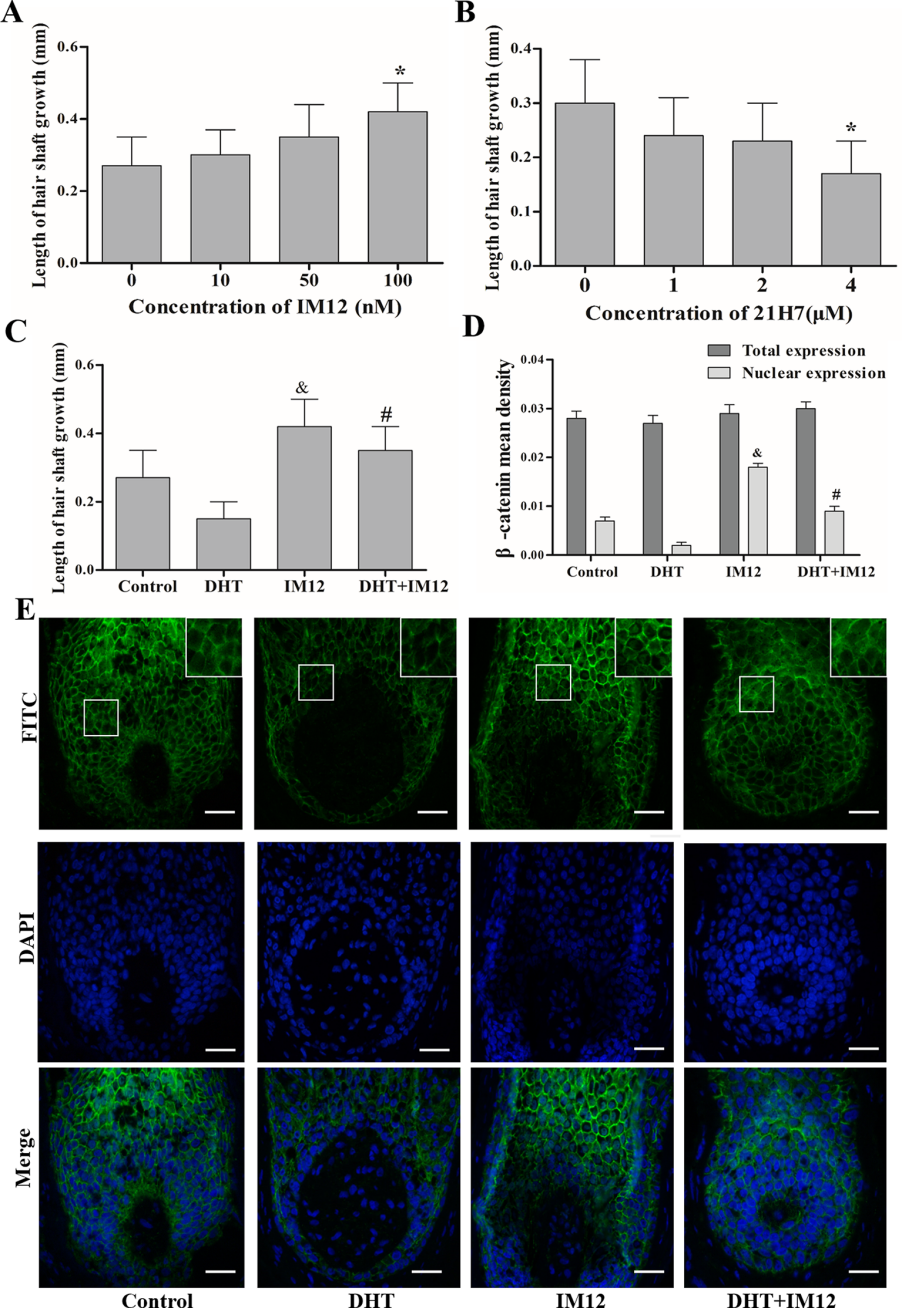
Dihydrotestosterone (DHT) regulates human hair follicles (HFs) growth through the Wnt/β-catenin pathway. HFs were cultured with IM12 (50, 100, or 500 nM), 21H7 (1, 2, or 4 µM), DHT (10^-6^ mol/L), IM12 (500 nM), or 10^-6^ mol/L DHT and 500 nM IM12. **(A**, **B)** Mean growth rates of hair shafts treated with IM12 and 21H7 (n = 10). **(C)** Mean growth rates of hair shafts treated with DHT and IM12 (n = 10). **(D**, **E)** Immunofluorescence staining and mean fluorescence intensity of β-catenin in HFs cultured with DHT and IM12 (n = 6). Magnification: 200× and 400×. The data are presented as the mean ± SD. Each bar represents the mean of three independent experiments performed in triplicate. Compared with the control (0 ) group, *P < 0.05 , *^&^P* < 0.05 and ^#^*P* < 0.05. Scale bar 50 μm (200×).

**Figure 7:**
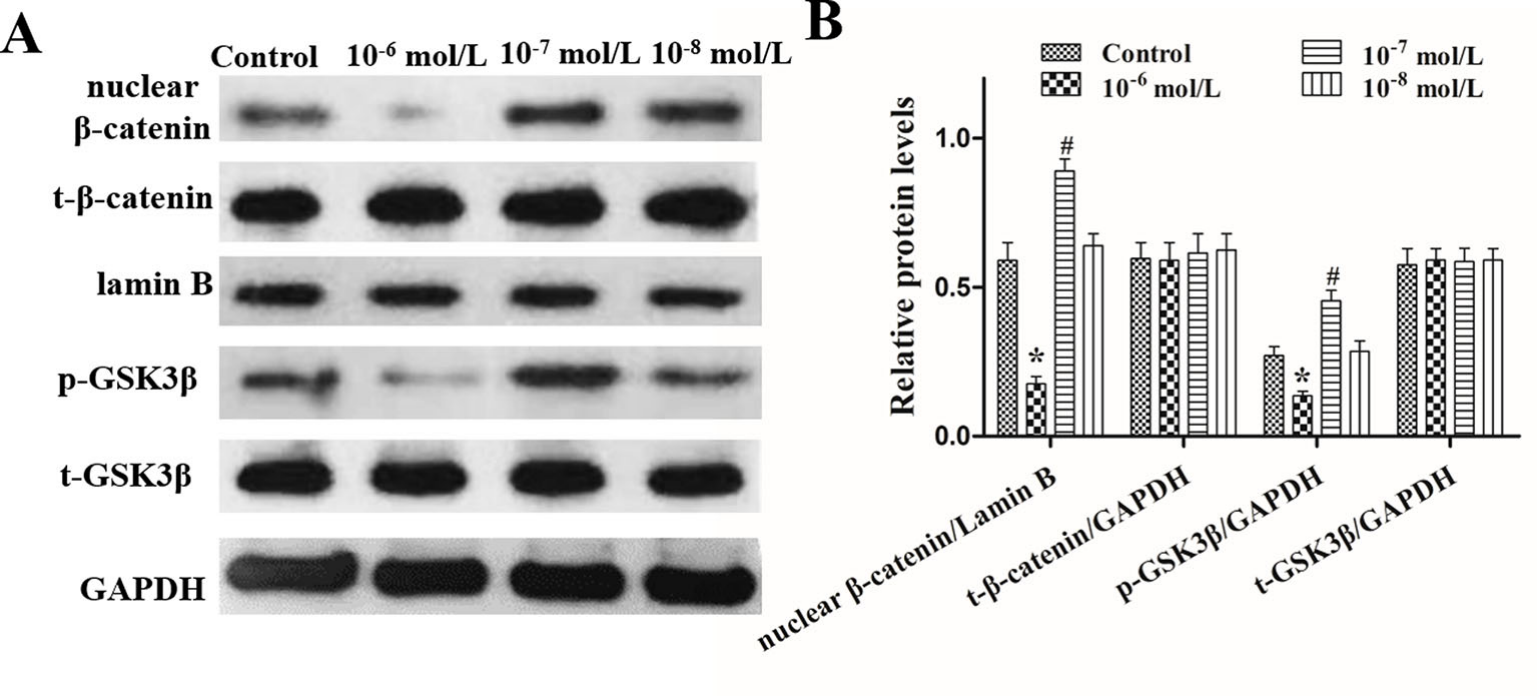
Effects of different concentrations of dihydrotestosterone (DHT) on the expression of β-catenin, GSK3β, and p-GSK3β (ser9) in C57BL/6 mice. The nucleoproteins and total proteins of the back skin were extracted for Western blot. **(A)** Western blot analysis of the expression of nuclear β-catenin, total β-catenin (t-β-catenin), total GSK3β (t-GSK3β), and p-GSK3β (ser9) (p-GSK3β) in the back skin. **(B)** The statistical results of Western blot analysis. The data are presented as the mean ± SD (n = 5). Each bar represents the mean of three independent experiments performed in triplicate. Compared with the control group, ^*^P < 0.05 and ^#^*P* < 0.05.

The effects of DHT on β-catenin, GSK3β, and p-GSK3β (ser-9) levels in cultured human HFs and mouse HFs were analyzed using semi-quantitative fluorescence and Western blot. In human HFs, we found that high concentrations of DHT (10^-5^ and 10^-6^ mol/L) failed to induce the phosphorylation of GSK3β at Ser-9 and thus inhibited β-catenin translocation into the nucleus, while low concentrations of DHT (10^-7^ mol/L) induced the phosphorylation of GSK3β at Ser-9 and thereby promoted the nuclear translocation of β-catenin (**Figure 4**). Immunofluorescence staining showed that AR is mainly expressed in dermal papilla cells and hair matrix cells (**Figure 5**). In addition, different concentrations of DHT had no effect on GSK3β expression (*P* > 0.05). Furthermore, HFs growth was inhibited by 21H7 and induced by IM12 in a dose-dependent manner, and IM12 promoted the growth of HFs treated with 10^-6^ mol/L DHT (**Figures 6A–C**). In addition, IM12 (500 nM) promoted β-catenin translocation into the nucleus and antagonized the inhibitory effect of DHT (10-6 M) on β-catenin nuclear localization (**Figures 6D, E**). In mouse HFs, we found that 10^-6^ mol/L DHT reduced the phosphorylation of GSK3β at Ser-9 and thus inhibited β-catenin translocation into the nucleus, while 10^-7^ mol/L DHT induced the phosphorylation of GSK3β at Ser-9 and thereby promoted the nuclear translocation of β-catenin (**Figure 7**). In addition, different concentrations of DHT had no effect on total GSK3β and total β-catenin expression (*P* > 0.05). These results suggest that DHT affects HF growth through the Wnt/β-catenin pathway.

## Discussion

Studies have found that 5αR2 is more highly expressed in the AGA balding scalp and beard HFs in males than in nonbalding AGA scalps (Price, 1999), so we speculated that the region-specific expression of 5αR2 induces the distinct effects of androgens on HF growth. However, it is difficult to assess the influence of 5αR2 on HF growth because 5αR cannot be maintained in culture systems *in vitro*. Therefore, we used DHT in our study. We found that DHT at concentrations of 10^-5^ mol/L and 10^-6^ mol/L significantly inhibited HF growth, while 10^-7^ mol/L DHT promoted HF growth compared with 10^-8^ mol/L DHT. Moreover, the effect of DHT on hair growth in C57BL/6 mice is similar to that on *in vitro* HFs. These results suggest that different concentrations of DHT greatly contribute to androgen-induced HF development. Many scholars have demonstrated that the scalp skin DHT concentration in AGA is significantly higher than that in hair-containing scalp (Price, 1999), which is consistent with the results of our experiments showing that high-dose DHT inhibits HF growth. However, after treatment with finasteride, the scalp skin DHT levels decreased, and AGA progression was delayed (Bang et al., 2004; Buchanan and Robaire, 2010; Prahalada et al., 2015). In our study, we found a similar phenomenon: when the DHT concentration decreased from 10^-6^ mol/L to 10^-7^ mol/L, the HFs grew much better than in the presence of higher DHT concentrations. In fact, the results suggest that an appropriate level of DHT is required for normal androgen-sensitive HF growth. Once the DHT concentration decreased from 10^-7^ mol/L to 10^-8^ mol/L, the HF growth rate showed no significant difference from that in the control group, which explains why beard growth is weaker in castrated males.

Activation of the Wnt/β-catenin pathway is important for HF regeneration and hair shaft growth. It has been reported that HFs cannot form when β-catenin, a key signaling molecule in this pathway, is mutated in the hair substrate of mice. When the mutant is induced in normal mice, the HFs do not enter the next hair growth cycle (Huelsken et al., 2001). Moreover, the loss of β-catenin activity directly leads to a block in the differentiation of HFSCs into HF cells (Povelones and Nusse, 2002; Furlong, 2005). Thus, we concluded that DHT affects HF growth differently by regulating the translocation of β-catenin, which is mediated by GSK3β. GSK3β activity is determined by several factors, and it has been demonstrated that phosphorylation at ser-9 is essential for decreasing GSK3β activity (Shimizu and Morgan, 2004; Mulholland et al., 2005). As expected, the Wnt/β-catenin pathway is negatively influenced by high levels of DHT (10^-5^ and 10^-6^ mol/L) in cultured HFs. Our results are in agreement with those of Leirós GJ et al., who found that DHT can downregulate the expression of p-GSK3β (ser9) and β-catenin in HFs from AGA patients (Leirós et al., 2012). Notably, HFs treated with 10^-7^ mol/L DHT showed an obvious increase in the nuclear expression of β-catenin in the hair matrix. Therefore, we hypothesized that 10^-7^ mol/L DHT promotes HF growth by activating the Wnt/β-catenin signaling pathway, while high levels of DHT have the opposite effect. In the normal hair follicle cycle, immunofluorescence staining indicated that AR is expressed in the dermal papilla cells and hair matrix cells, so we speculate that DHT might regulate Wnt/β-catenin signaling pathway by binding with AR in these two types of cells. However, the exact mechanism needs to be further confirmed.

To further demonstrate the role of the Wnt signaling pathway in HF growth, we used IM12 and 21H7 to mimic the effects of activating and inhibiting the Wnt signaling pathway (Schm?Le et al., 2010; Tsai et al., 2014). In the presence of IM12, a β-catenin-specific activator that impacts GSK3β, the growth rate of HFs *in vitro* was significantly accelerated. Overwhelming evidence has shown that the activation of β-catenin is necessary to induce HFs (Celso et al., 2004). However, the addition of the β-catenin inhibitor 21H7 markedly inhibited the growth of HFs. David Enshell-Seijffers et al. also found that ablation of β-catenin in HFs results in dramatic hair shortening and thinning (Enshell-Seijffers et al., 2010). A similar positive effect of IM12 on HFs was observed upon cotreatment with 10^-6^ mol/L DHT. Considering both of these results, it is conceivable that DHT is involved in regulating HF growth by modulating the translocation of β-catenin to the nucleus. From our results, we conclude that the effects of DHT are closely related to its concentration; actually, DHT can activate the Wnt pathway at an appropriate concentration.

Functional cross-talk between the androgen and Wnt/β-catenin signaling pathways has been described in this study. Our results indicate that DHT can regulate the expression of molecules necessary for HF development, and some of these factors involved in androgen-induced hair growth are encoded by target genes of the Wnt/β-catenin pathway. We found that an appropriate concentration of DHT resulted in an increase in p-GSK3β (ser-9) levels in cultured HFs, followed by β-catenin translocation into the nucleus and activation of the transcription of downstream target genes (here means activate Wnt/β-catenin pathway), which prompted faster HF growth. As shown in our study, high-dose DHT had a negative effect on HFs that was diminished by cotreatment with a β-catenin activator.

Previous studies have found that androgen has different effects on hair follicles in different regions, and appropriate concentrations of androgens can promote hair growth, while excessive concentrations of androgens can inhibit hair growth (Itami and Inui, 2005). Dermal papilla cells (DPC) can induce hair follicle stem cells (HFSC) to differentiate into hair follicles, while DHT inhibits HFSC differentiation by interfering with Wnt pathway in a coculture model with human DPC and HFSC (Leirós et al., 2012; Leirós et al., 2017). Of course, these studies have given us a much clearer understanding of the growth of hair follicles, but we also have found that these studies have not been fully verified from the organ level. Therefore, this study verified the effects of different concentrations DHT on the hair follicle, and found that the expression levels of Wnt pathway protein (β-catenin) changed significantly in DHT cultured hair follicles, and the activator of Wnt pathway (IM12) antagonized the inhibition of high concentration DHT on hair follicle growth *in vitro*, and further confirmed that the growth of hair follicles was indeed regulated by androgen and Wnt/β-catenin pathway at the organ level.

Unfortunately, our experiments have not found the cause of the regional effects of DHT on HFs. As mentioned above, the concentration of DHT explains these differences, and AR is also involved in the development of AGA, so there may be some unknown mechanisms which regulate the growth of HF. Therefore, in the coming studies, it’s necessary to elucidate the mechanism by which DHT regulates GSK3β activity, and which type of DHT target cells in the hair follicle will be, and what pathway of DHT could activate or inhibit the Wnt pathway. Until now, according to previous studies and our findings, we infer that the kinases that participate in phosphorylating GSK3β at ser-9 may be target genes of DHT.

## Data Availability Statement

The raw data supporting the conclusions of this article will be made available by the authors, without undue reservation, to any qualified researcher.

## Ethics Statement

The Institutional Review Boards of all participating centers approved the protocol used in this study, and all procedures were performed in accordance with the ethical standards established in the Declaration of Helsinki. All volunteers provided written informed consent before starting the study. The experimental studies were approved by the Institutional Animal Care and Use Committee of The Third Affiliated Hospital of Sun Yat-sen University (Approval No: SYU20180502).

## Author Contributions

MW and XC designed the experiments. XC and BL wrote the manuscript. XC, YL, XT, and LH performed experiments. WD and WL analyzed the data. All authors have read and approved the final manuscript.

## Funding

This work was supported by the National Natural Science Foundation of China (No. 81271769 and 81872542).

## Conflict of Interest

The authors declare that the research was conducted in the absence of any commercial or financial relationships that could be construed as a potential conflict of interest.
